# Association between diaphragm thickness and postoperative complications in elderly patients with non-small-cell lung cancer

**DOI:** 10.1007/s00595-025-03130-x

**Published:** 2025-09-20

**Authors:** Shoji Kuriyama, Motoko Konno, Naoko Mori, Sumire Shibano, Shinogu Takashima, Tsubasa Matsuo, Yusuke Sato, Kyoko Nomura, Yoshihiro Minamiya, Kazuhiro Imai

**Affiliations:** 1 Department of Thoracic Surgery, Akita University Graduate School of Medicine, 1-1-1 Hondo, Akita, 010-8543 Japan; 2 Department of Radiology, Akita University Graduate School of Medicine, 1-1-1 Hondo, Akita, 010-0853 Japan; 3 Department of Health Environmental Science and Public Health, Akita University, 1-1-1 Hondo, Akita, 010-0853 Japan

**Keywords:** Non-small-cell lung cancer, Elderly patients, Computed tomography, Diaphragm, Postoperative complications

## Abstract

**Purpose:**

Predicting perioperative complications in high-risk elderly patients with lung cancer has become increasingly important as the population ages. This study investigated the relationship between preoperative diaphragmatic thickness (DT) and perioperative complications.

**Methods:**

We enrolled 101 patients ≥ 75 years old who had undergone radical resection for primary lung cancer between 2013 and 2018. Bilateral DT was measured on axial and coronal computed tomography, and the mean DT (MDT) was calculated based on these measurements. Outcomes were assessed based on postoperative complications, defined as Clavien–Dindo classification ≥ 2.

**Results:**

The MDT was 3.51 ± 1.00 mm. Thirteen patients who experienced postoperative respiratory complications had a significantly lower MDT than a higher MDT (*p* = 0.0390). Multivariate logistic regression analyses revealed that an MDT ≤ 3.63 mm was an independent factor associated with postoperative complications (odds ratio, 5.559).

**Conclusions:**

Patients with a low MDT are at an increased risk of postoperative complications. Therefore, these patients require careful perioperative management.

**Supplementary Information:**

The online version contains supplementary material available at 10.1007/s00595-025-03130-x.

## Introduction

Lung cancer remains the leading cause of cancer-related mortality [1], and surgical resection is the most curative treatment for early stage non-small-cell lung cancer (NSCLC) [2]. There is an increasing number of elderly patients undergoing surgery for NSCLC [3, 4], which places this population at a high risk of decreased physical capacity and increased comorbidities.

Sarcopenia is a syndrome characterized by progressive and generalized loss of skeletal muscle mass and strength, resulting in physical disability, a poor quality of life, and death [5]. Sarcopenia adversely affects the outcome of patients with various forms of cancer [6]. In elderly patients with NSCLC who have undergone surgery, sarcopenia is associated with a poor prognosis and a high risk of postoperative complications [7, 8].

Respiratory sarcopenia is a condition in which both low respiratory muscle strength and low respiratory muscle mass are present [9]. The strength of the respiratory muscles was assessed using voluntary respiratory muscle strength tests, including maximum inspiratory/expiratory mouth pressures (MIP/MEP). However, the volume of the respiratory muscles, such as the diaphragm, intercostal muscles, and pectoralis major, is commonly evaluated using ultrasonography and computed tomography (CT). However, the postoperative impact on the respiratory muscle volume and function in patients with NSCLC remains unclear.

In the present study, we focused on the diaphragm, the primary inspiratory muscle, to investigate the association between preoperative diaphragm thickness (DT) on CT and postoperative respiratory complications and outcomes in elderly patients with NSCLC who had undergone pulmonary resection.

## Methods

### Patients and surgical procedures

**Fig. 1:**
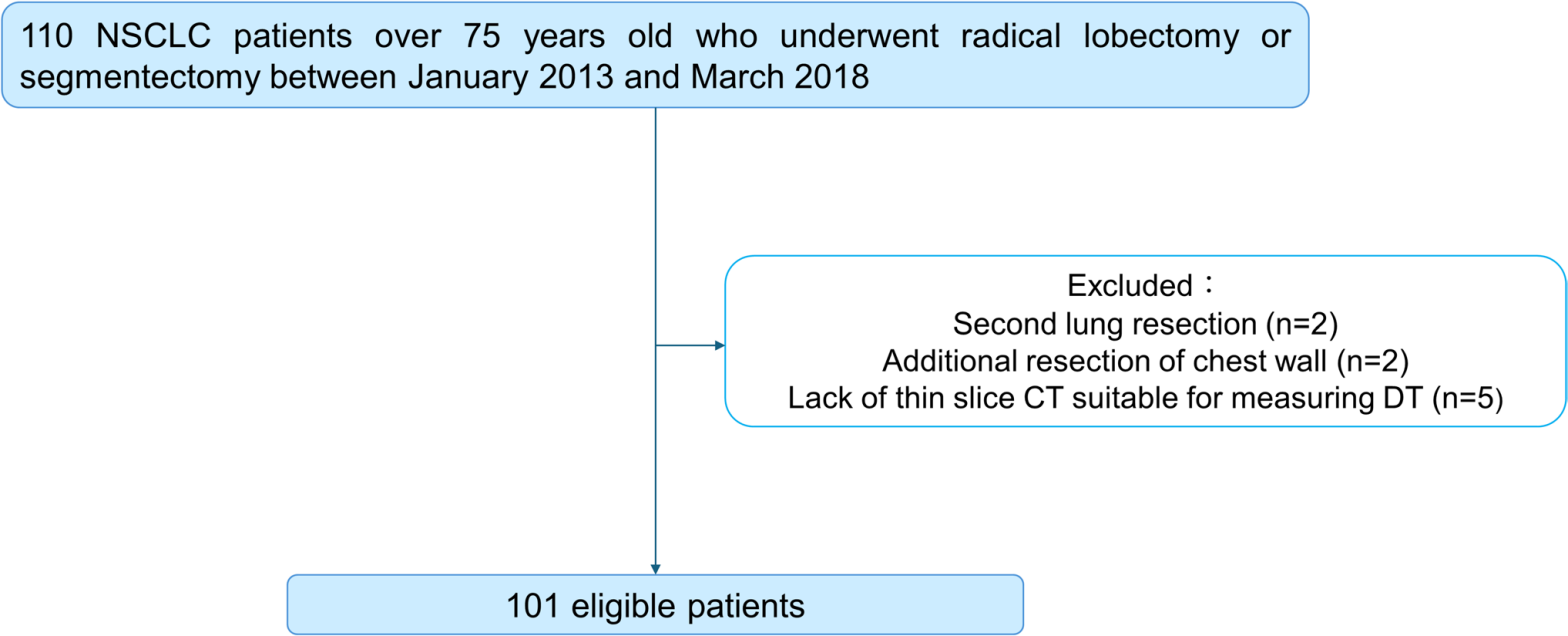
Flow chart illustrating the subject enrollment protocol

The study protocol was reviewed and approved by the institutional review board (IRB) of Akita University Hospital [approval number: 2679, date of June 28, 2021], and all medical records were collected under the IRB-approved protocol. The need for individual patient consent was waived because all patient data were anonymized. We enrolled 101 patients ≥ 75 years old who had undergone radical lobectomy or segmentectomy for NSCLC between January 2013 and March 2018 at Akita University Hospital. All patients received standard preoperative and intraoperative care. The surgical procedure was determined based on patient tolerance and tumor characteristics. Postoperative respiratory complications observed within 30 days after surgery were classified according to the Clavien–Dindo classification [10]. Patient characteristics and detailed postoperative complications are listed in Table 1. Chronic obstructive pulmonary disease (COPD) was defined as FEV1% < 70%. A diagram illustrating the process by which the cases were selected for this study is shown in Fig. 1.

**Table 1:** Patient characteristics and postoperative respiratory complications

	N = 101
Age (years)	78.7 ± 2.8
Gender (Male/Female)	62 (61.4%)/39 (38.6%)
Performance status (0/≥ 1)	81 (80.2%)/20 (19.8%)
COPD	28 (27.7%)
Charlson comorbidity index (0,1/≥ 2)	67 (66.3%)/34 (33.7%)
Pulmonary function tests	
VC (L)	2.82 ± 0.61
%VC (%)	106.7 ± 15.0
FEV1 (L)	2.04 ± 0.47
%FEV1 (%)	75.5 ± 10.7
Location (Right/Left)	55 (54.5%)/46 (45.5%)
Operative Procedures (Lob./Seg.)	77 (76.2%)/24 (23.8%)
Operation Time (min)	217 ± 48
Tumor subtype (AD/SCC/Others)	76 (75.2%)/25 (24.8%)/0 (0%)
Tumor size (mm)	26.3 ± 10.3
Pathological Stage (IA/IB/IIA/IIB/IIIA)	58 (56.9%)/23 (22.8%)/8 (7.9%)/9 (8.9%)/3 (3.0%)
Postoperative respiratory complications (CD grade ≥ II)	13 (12.9%)
Pleural leakage	7 (6.9%)
Pneumonia	3 (3.0%)
Atelectasis	1 (1.0%)
Interstitial pneumonia	1 (1.0%)
Pneumothorax	1 (1.0%)

*COPD* chronic obstructive pulmonary disease, *VC* vital capacity, *FEV1* forced expiratory volume in one second, *CD* Clavien–Dindo classification

### Measurement of the DT

**Fig. 2:**
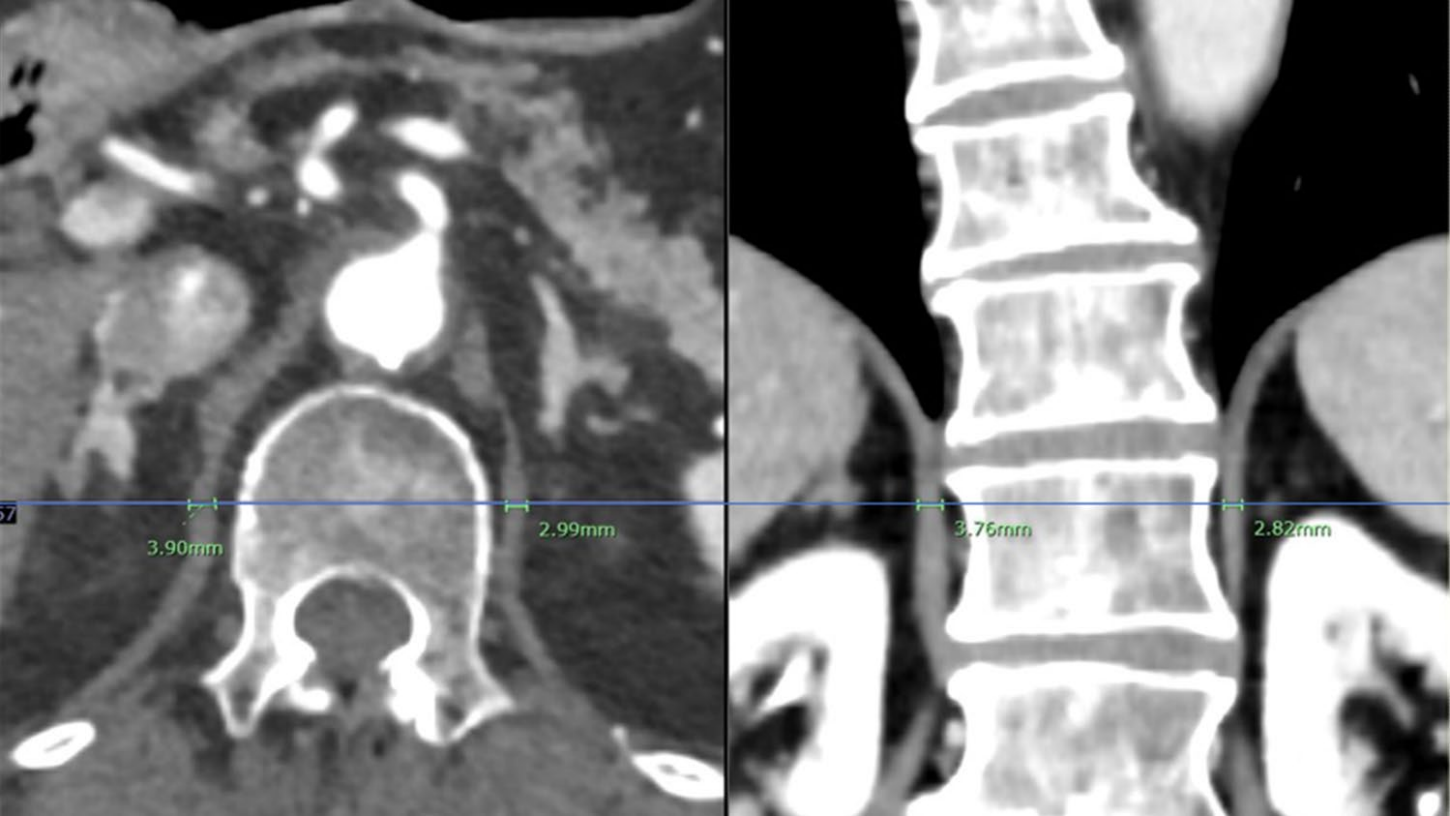
Measurement of both hemidiaphragm thicknesses at the middle vertebral body at the level of the celiac artery origin on axial and coronal CT

Before surgery, all patients underwent contrast-enhanced chest CT (Discovery CT750 HD or Revolution CT; GE HealthCare Technologies Inc., Chicago, United States) while they were in a supine position at end-inspiration. Reformatted images were acquired with a slice thickness of 1.25 mm in both axial and coronal planes. In axial CT images, the thickness of both hemidiaphragms was measured at the level of the middle vertebral body at the origin of the celiac arterial trunk. The thickness of the corresponding hemidiaphragms was also measured on coronal sections [11–13]. The measurements of each patient were performed by two thoracic surgeons (S.K. and S.S., with 12 and 4 years of thoracic surgical experience, respectively; Fig. 2), after which the mean value obtained from these measurements was defined as the mean DT (MDT) for each patient.

### Statistical analyses

Group data are expressed as the mean ± standard deviation. The reliability of the measurement by the two observers was evaluated using intraclass correlation coefficient (ICC) scores with a 95% confidence interval (CI). The ICC was calculated using the following formula with a two-way random effects model (absolute agreement, multiple raters/measurement) to quantify the level of consistency and agreement.

ICC values were interpreted according to the guidelines proposed by Koo and Li, as follows [14]: < 0.50, poor reliability; 0.50–0.75, moderate reliability; 0.75–0.90, good reliability; > 0.90, excellent reliability. Receiver operating characteristic (ROC) curves and Youden's index [15] were used to determine the cutoff value that yielded the highest combined sensitivity and specificity with respect to the likelihood of experiencing postoperative complications. The areas under the ROC curves (AUCs) were calculated using standard formulae for the collected data. The relationship between MDT and confounders affecting the apparent likelihood of postoperative complications was assessed using univariate and multivariate logistic regression analyses. The variables in the multivariate logistic regression analysis were selected using a stepwise method. A Kaplan–Meier analysis was used to estimate the overall survival (OS), and the curves were compared using the log-rank test. P values were 2-sided and considered significant if p < 0.05. Statistical analyses were performed using the JMP IN 17.2.0 software program (SAS Institute, Cary, NC, USA).

## Results

**Fig. 3:**
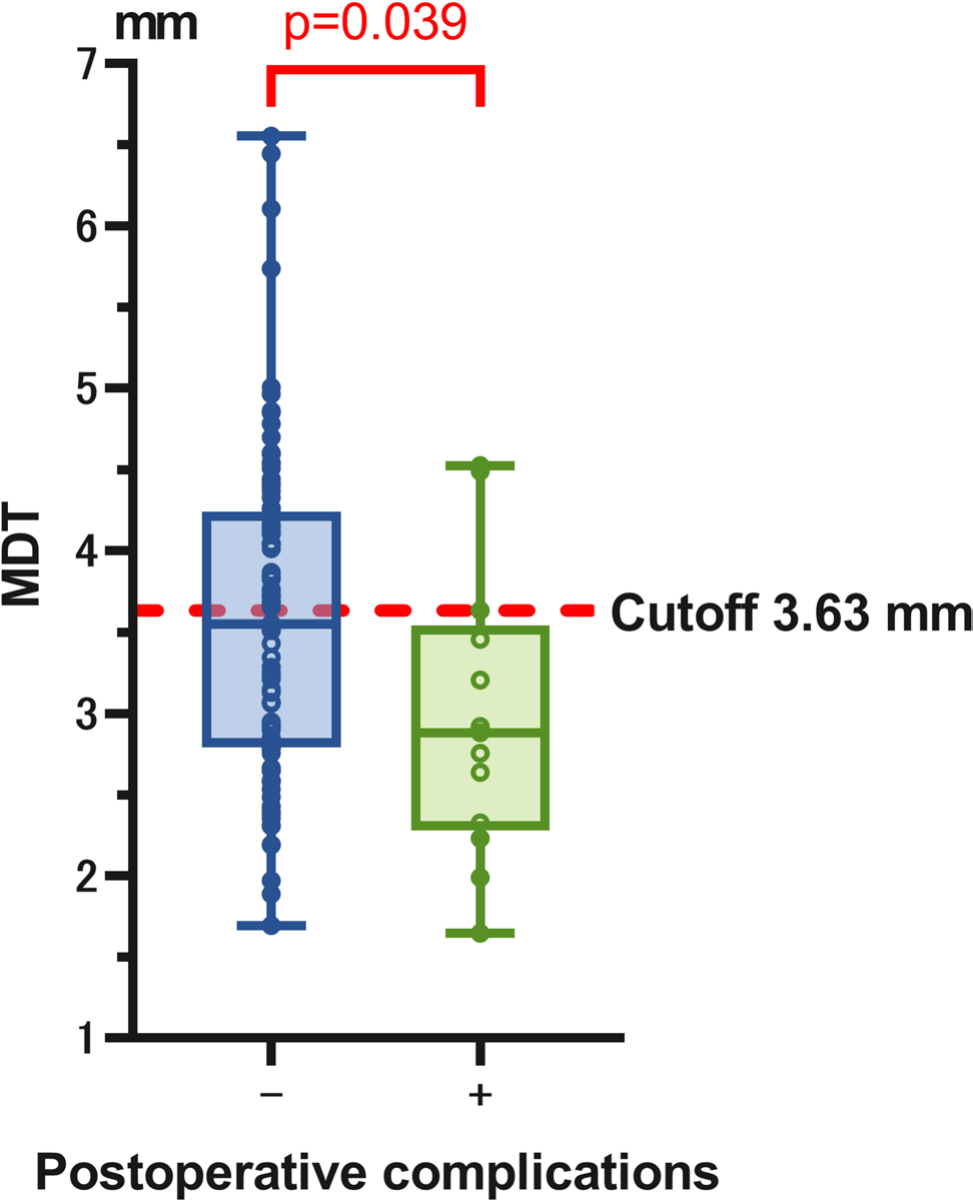
The comparison of MDTs between patients with and without postoperative complications

Table 2 shows the results of each DT measurement for the 101 patients between the 2 observers. The ICC between the observers ranged from 0.6051 to 0.7473, suggesting substantial agreement. The MDT was significantly lower in patients who experienced postoperative complications, defined as Clavien–Dindo classification ≥ 2 (p = 0.0390, Fig. 3), than in those who did not. To determine the cutoff values that yielded the highest combined sensitivity and specificity for predicting postoperative complications, a conventional ROC curve was used to analyze the MDT (Supplementary Fig. 1). The optimal MDT cutoff value was calculated to be 3.63 mm (AUC = 0.6788). There was no significant correlation between the preoperative pulmonary function and MDT (Supplementary Fig. 2). No significant correlation was observed between the MDT and psoas muscle cross-sectional area at the L3 level on CT (Supplementary Fig. 3). Of the three patients who underwent reoperation, two exhibited a reduction in diaphragm muscle thickness postoperatively. To evaluate potential confounding factors, we compared the baseline patient characteristics between the two groups divided by the MDT cutoff value (3.63 mm). As shown in Supplementary Table 1, there were no significant differences in any of the characteristics between the high- and low-MDT groups.

**Table 2:** CT measurements and inter-observer agreement (N = 101)

	Observer 1	Observer 2	ICC
Right DT on axial (mm)	3.91 ± 1.40	3.98 ± 1.56	0.7311
Left DT on axial (mm)	3.39 ± 1.34	3.43 ± 1.27	0.6051
Right DT on coronal (mm)	3.63 ± 1.30	3.65 ± 1.31	0.6587
Left DT on coronal (mm)	3.03 ± 1.08	3.01 ± 1.16	0.6247
Mean DT (mm)	3.49 ± 1.03	3.52 ± 1.10	0.7473

*DT* diaphragm thickness, *ICC* intraclass correlation coefficient, *CT* computed tomography

The ICC was calculated using a two-way random effect model, absolute agreement, multiple raters/measurements

**Fig. 4:**
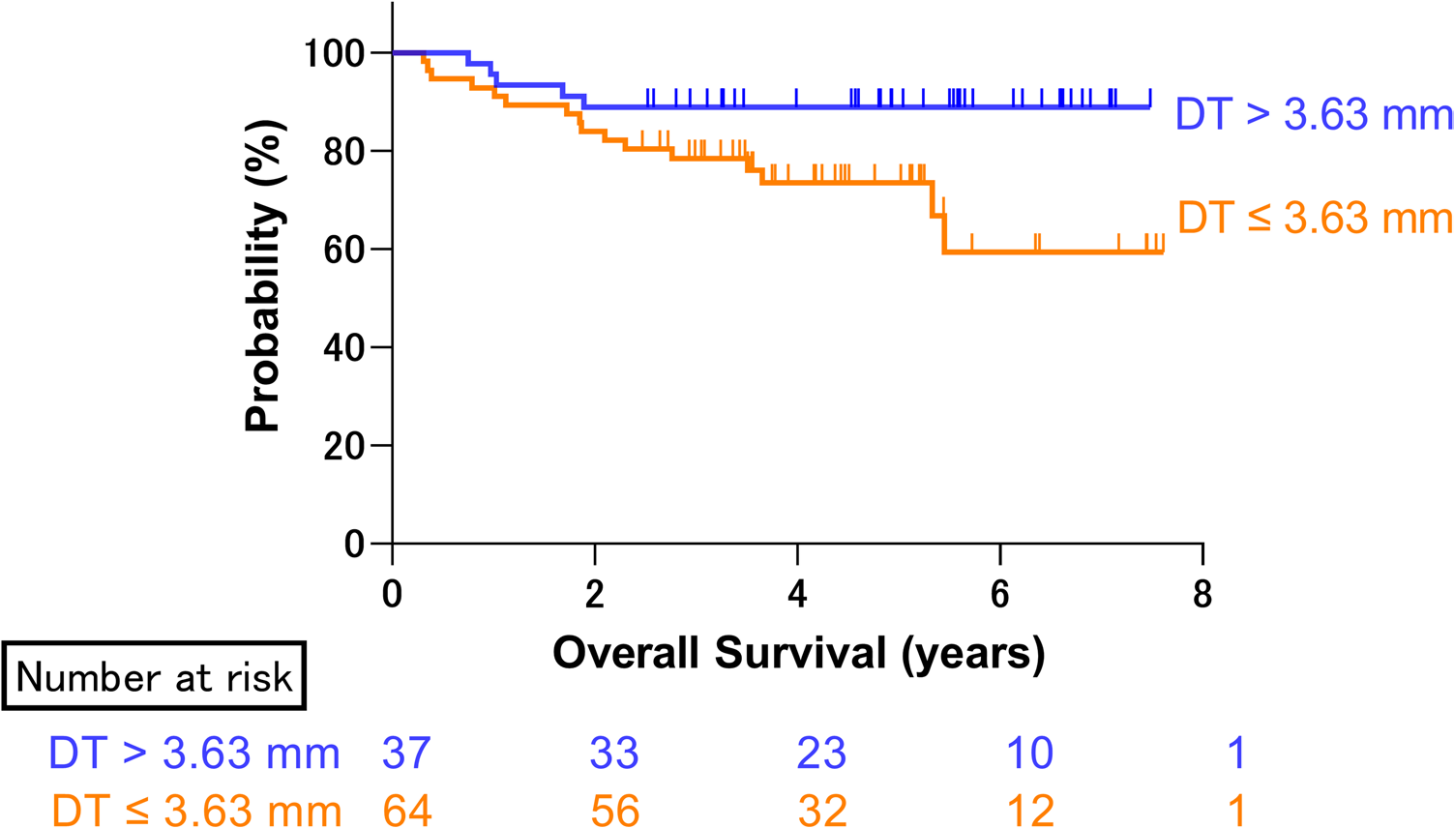
The comparison of the overall survival (OS) between patients divided based on a cutoff MDT of 3.63 mm

Age (> 80 or ≤ 80 years old), sex (male or female), performance status (PS) (0 or ≥ 1), COPD (presence or absence), Charlson comorbidity index (CCI) (≥ 2 or 0, 1), surgical procedure (segmentectomy or lobectomy), operation time (> 210 or ≤ 210 min), and MDT (> 3.63 or ≤ 3.63 mm) were evaluated using univariable and multivariable logistic regression analyses. The MDT (odds ratio [OR], 5.559; 95% CI, 1.121–27.565) was an independent risk factor for postoperative complications in the univariable and multivariable models (Table 3). The Kaplan–Meier analysis showed a poor prognosis for patients with MDT ≤ 3.63 mm compared to those with MDT > 3.63 mm (log-rank p = 0.0263, Fig. 4). In the univariate analysis, pathological stage and low MDT were independent predictors of a poor prognosis; however, in the multivariate analysis, only pathological stage remained an independent prognostic factor (Supplementary Table 2).

**Table 3:** Univariable and multivariable analyses for postoperative respiratory complications (logistic regression analyses)

	Univariable analysis				Multivariable analysis			
	95% CI		*P*	Odds ratio	95% CI		*P*	Odds ratio
Age (> 80 years old)	0.095	2.216	0.3220	** − **	** − **	** − **	** − **	** − **
Gender (male)	0.834	19.083	0.0653	** − **	0.5401	15.236	0.2161	−
PS (≥ 1)	0.311	5.053	0.7509	** − **	−	−	−	−
COPD	0.524	5.949	0.3541	−	−	−	−	−
Charlson comorbidity index (≥ 2)	0.809	8.585	0.0990	−	0.698	8.694	0.1610	−
Procedure (segmentectomy)	0.665	7,745	0.1822	−	−	−	−	−
Operation time (> 210 min)	0.859	12.934	0.0693	−	0.586	10.721	0.2151	−
MDT (≤ 3.63 mm)	1.100	25.099	0.0234*	5.256	1.138	28.430	0.0342*	5.688

^*^ significant difference

*COPD* chronic obstructive pulmonary disease, *MDT* mean diaphragm thickness, *CI* confidence interval

## Discussion

In the present study, we observed that MDTs measured on CT were associated with postoperative complications in elderly patients with NSCLC. Furthermore, a univariable/multivariable logistic regression analysis showed that MDT ≤ 3.63 mm was an independent risk factor for postoperative complications.

The incidence of postoperative complications following pulmonary resection for NSCLC ranges from approximately 13% to 39% [16–20], with higher rates among elderly patients [17, 19, 21]. Because postoperative complications are reportedly associated with the long-term prognosis [16, 22], the ability of thoracic surgeons to predict high-risk patients to minimize incidence rates is highly important. Comorbidities are known risk factors [23–26], and the CCI serves as a useful predictor of postoperative outcomes [27]. Other risk factors include male sex [28], a smoking history or COPD [17, 29, 30], PS [19, 31], and the prognostic nutrition index [32, 33]. In the present study, univariate and multivariate logistic regression analyses employed these covariates, and a low MDT emerged as a significant risk factor in the multivariate analysis.

Previous reports on DT using CT have shown MDT values ranging approximately from 3.3 to 4.3 mm, with the right side being thicker than the left. These findings are consistent with the results of the present study. Furthermore, a relationship between DT and the age, sex, body mass index, and skeletal muscle mass has been reported [34]. The DT has been shown to correlate positively with appendicular skeletal muscle mass, handgrip strength, and gait speed in older adults [35]. The association between the MDT and postoperative complications may be explained by the physiological role of MDT. As the primary inspiratory muscle, the diaphragm is essential for effective ventilation, airway clearance, and coughing [36]. A thinner diaphragm indicates reduced muscle strength, leading to ineffective airway mucus clearance and an increased susceptibility to pneumonia and atelectasis. In addition, several studies have reported sarcopenia as a risk factor for poor wound healing [37, 38]. Since MDT is also a surrogate marker of whole-body sarcopenia, a low MDT may indirectly affect delayed healing of pulmonary fistula.

Sarcopenia is reported to put patients at a high risk for morbidity and poor prognosis [6–8]. However, respiratory sarcopenia is a relatively new concept, defined as a reduction in respiratory muscle strength and mass, and its impact of respiratory sarcopenia on lung cancer patients remains unclear. This ambiguity arises because the methods and criteria for diagnosing respiratory sarcopenia vary and have not yet been standardized. Respiratory muscle strength is primarily evaluated using MIP/MEP, but the range varies among studies [39], in part because the measurements depend on the spirometer used and shape of the mouthpiece [40]. The respiratory muscles that can be assessed for muscle mass include the diaphragm [41, 42], intercostal muscles [43], and accessory respiratory muscles, such as the pectoral muscles [44] and erector spinae [45], although there is currently no consensus on the optimal method for this assessment.

The present study focused on the diaphragm, the major inspiratory muscle, to assess the respiratory muscle mass. Our findings suggest that, when treating elderly patients with NSCLC, these measurements can help identify patients at an increased risk of postoperative complications. In clinical practice, elderly patients with a low MDT may benefit from individualized perioperative strategies. For example, patients identified as being at high risk based on a reduced MDT could be considered for respiratory training, including airway clearance techniques to prevent postoperative pneumonia and atelectasis. In addition, more careful intraoperative precautions to prevent pulmonary fistula, close postoperative monitoring, and less invasive surgical approaches, such as wedge resection, when oncologically appropriate, may also be advisable. However, whether or not such interventions are truly effective in elderly lung cancer patients with a low MDT remains to be determined, and further large-scale prospective studies are warranted.

CT and ultrasonography are commonly used to evaluate the diaphragm muscle. CT, which is routinely performed before lung resection, noninvasively provides an objective assessment based on high-resolution imaging. Ultrasonography enables the evaluation of muscle dynamics, such as thickening fraction and excursion due to respiration, and is noninvasive, minimizing patient burden. This study adopted a CT-based assessment method that allowed retrospective data collection. This method is convenient, quick, and can help to identify high-risk patients.

Several limitations associated with the present study warrant mention. First, it had a retrospective, single-center design and a small sample size. Second, this method may have inter-observer variability. Since diaphragmatic muscle thicknesses measured on CT are extremely thin (1–9 mm) and uneven, there may have been some measurement discrepancies between observers. The cutoff value identified in this study should be interpreted with caution. However, in the present study, two observers performed the DT measurements, and the inter-observer variability was validated. The ICC values suggested moderate reliability. Further research is needed to validate the optimal cutoff values and measurement errors, although our findings suggest that this method may be acceptable for predicting which elderly patients with NSCLC are at a higher risk.

## Conclusions

The incidence of postoperative complications was significantly higher in elderly NSCLC patients with an MDT of ≤ 3.63 mm measured using a preoperative CT than > 3.63 mm. This suggests that MDT measurements can serve as a guide for selecting appropriate therapeutic strategies for high-risk patients.

## Supplementary Information

Below is the link to the electronic supplementary material.

Supplementary file1 (DOCX 21 KB)

## Data Availability

The data supporting the findings of this study are available from the corresponding author upon reasonable request. **Institutional Review Board (IRB) Approval:** This retrospective study was reviewed and approved by the Institutional Review Board (IRB) at Akita University Hospital [approval number: 2679] on June 28, 2021.

## Abbreviations

AUC: Areas under curve
CCI: Charlson comorbidity index
CI: Confidence interval
COPD: Chronic obstructive pulmonary disease
CT: Computed tomography
DT: Diaphragm thickness
ICC: Intraclass correlation coefficient
IRB: Institutional review board
MDT: Mean diaphragm thickness
MIP/MEP: Maximum inspiratory/expiratory mouth pressures
NSCLC: Non-small-cell lung cancer
OR: Odds ratio
OS: Overall survival
PS: Performance status
ROC: Receiver operating characteristic
