# Supplementary material for: Association between diaphragm thickness and postoperative complications in elderly patients with non-small-cell lung cancer
Source: Surg Today. 2025 Sep 20;56(3):293–300. doi: 10.1007/s00595-025-03130-x (PMC12945973; doi:10.1007/s00595-025-03130-x)
Supplement: Supplementary file 1 — Supplementary file1 (DOCX 21 KB) [file 595_2025_3130_MOESM1_ESM.docx]

**Supplementary Table 1. Comparison of patient characteristics and postoperative complications between groups stratified by the MDT cutoff value**

|  | MDT > 3.63 (N = 45) | MDT ≤ 3.63 (N = 56) | *p* value |
| --- | --- | --- | --- |
| Age (year) | 78.3 ± 2.9 | 79.1 ± 2.7 | 0.091 |
| Gender (Male / Female) | 27 / 18 | 35 / 21 | 0.798 |
| Performance status (0 / ≥ 1) | 38 / 7 | 43 / 13 | 0.337 |
| COPD | 10 | 18 | 0.268 |
| Charlson comorbidity index (0,1 / ≥ 2) | 30 / 15 | 37 / 19 | 0.950 |
| Pulmonary function tests |  |  |  |
| VC (L) | 2.83 ± 0.54 | 2.82 ± 0.66 | 0.854 |
| %VC (%) | 108.2 ± 15.0 | 105.6 ± 15.1 | 0.367 |
| FEV1 (L) | 2.08 ± 0.43 | 2.01 ± 0.50 | 0.503 |
| %FEV1 (%) | 75.7 ± 8.0 | 75.3 ± 12.6 | 0.932 |
| Location (Right / Left) | 22 / 23 | 33 / 23 | 0.314 |
| Operative Procedures (Lob. / Seg.) | 36 / 9 | 41 / 15 | 0.424 |
| Operation Time (min) | 217 ± 43 | 216 ± 51 | 0.769 |
| Tumor subtype (AD / SCC / Others) | 35 / 10 | 41 / 15 | 0.596 |
| Tumor size (mm) | 24.3 ± 8.7 | 27.9 ± 11.3 | 0.219 |
| Pathological Stage (IA /IB /IIA /IIB /IIIA) | 28 / 12 / 2 / 3 / 0 | 30 / 11 / 6 / 6 / 3 | 0.335 |
| Postoperative respiratory complications  (CD ≥ grade II) | 2 | 11 | 0.023* |
| Pleural leakage | 1 | 6 |  |
| Pneumonia | 1 | 2 |  |
| Atelectasis | 0 | 1 |  |
| Interstitial pneumonia | 0 | 1 |  |
| Pneumothorax | 0 | 1 |  |

COPD: chronic obstructive pulmonary disease, VC: Vital capacity, FEV1: Forced expiratory volume in one second, CD: Clavien-Dindo classification

|  | Univariable analysis | | | | Multivariable analysis | | | |
| --- | --- | --- | --- | --- | --- | --- | --- | --- |
|  | 95% CI | | *P* | Hazard ratio | 95% CI | | *P* | Hazard ratio |
| Age (> 80 years old) | 0.430 | 2.861 | 0.8299 | **−** | **−** | **−** | **−** | **−** |
| Gender (male) | 0.662 | 4.411 | 0.2679 | **−** | − | − | − | − |
| PS (≥ 1) | 0.971 | 6.002 | 0.0578 | **−** | − | − | − | − |
| Charlson comorbidity index (≥ 2) | 0.814 | 4.518 | 0.1363 | − | − | − | − | − |
| pStage (≥ II) | 1.188 | 6.928 | 0.0191* | 2.869 | 1.018 | 6.050 | 0.0455* | 2.482 |
| MDT (≤ 3.63 mm) | 1.086 | 8.208 | 0.0340* | 2.986 | 0.955 | 7.403 | 0.0613 | − |

**Supplementary Table 2. Univariable and multivariable analysis for overall survival**

* significant difference
